# Di-μ-hydroxido-bis­[iodido­diphenyl­tin(IV)]–1,3-di­methyl­imidazolidin-2-one (1/2)

**DOI:** 10.1107/S2414314625007114

**Published:** 2025-08-21

**Authors:** Hans Reuter

**Affiliations:** a Chemistry, Osnabrück University, Barabarstr. 7, 49069 Osnabrück, Germany; Purdue University, USA

**Keywords:** crystal structure, diorganotin(IV)-hydroxide-halides, DMPU, hydrogen bonding

## Abstract

The title compound, di-μ-hydroxido-bis­[iodido­diphenyl­tin(IV)]–1,3-di­methyl­imidazolidin-2-one (1/2), [Sn(C_6_H_5_)_2_I(OH)]·2C_6_H_12_N_2_O, represents only the second example in the dimeric diorganotin(IV)-hydroxide-halide solvates [*R*_2_Sn(OH)*Hal*]_2_·2*BB* with *Hal* = I. As is usual for this class of compound, dimerization takes place *via* the oxygen atoms of the hydroxyl groups and leads to a planar, centrosymmetric, four-membered Sn—O ring of rhomboidal shape whose Sn—O distances [2.024 (2)/2.174 (2) Å] are determined by the position (*axial* or *equatorial*) of the oxygen atom on the respective trigonal–pyramidal coordinated tin atom while the bond angles are acute [70.74 (8)°] at the tin atoms and obtuse [109.26 (8)°] at the oxygen atoms.

## Abstract 2

In the solid, state di­phenyl­tin(IV)-hydroxide-iodide-*N*,*N*′-di­methyl­propyl­ene-urea, Ph_2_Sn(OH) ·DMPU, consists of centrosymmetric dimers exhibiting the characteristic structural features of diorganotin(IV)-hydroxide-halides.

## Graphical abstract

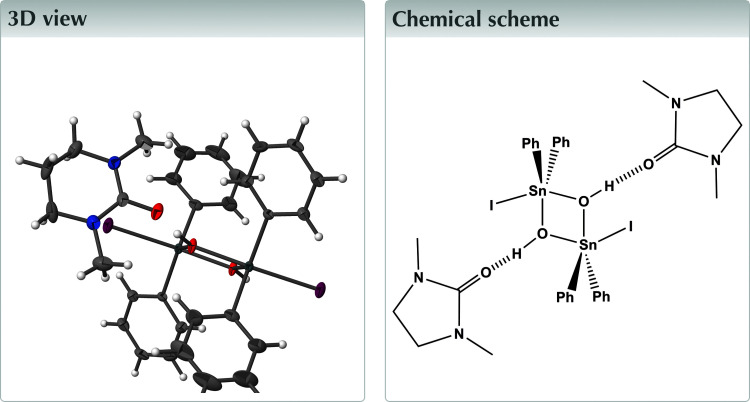

## Structure description

**Figure 1:**
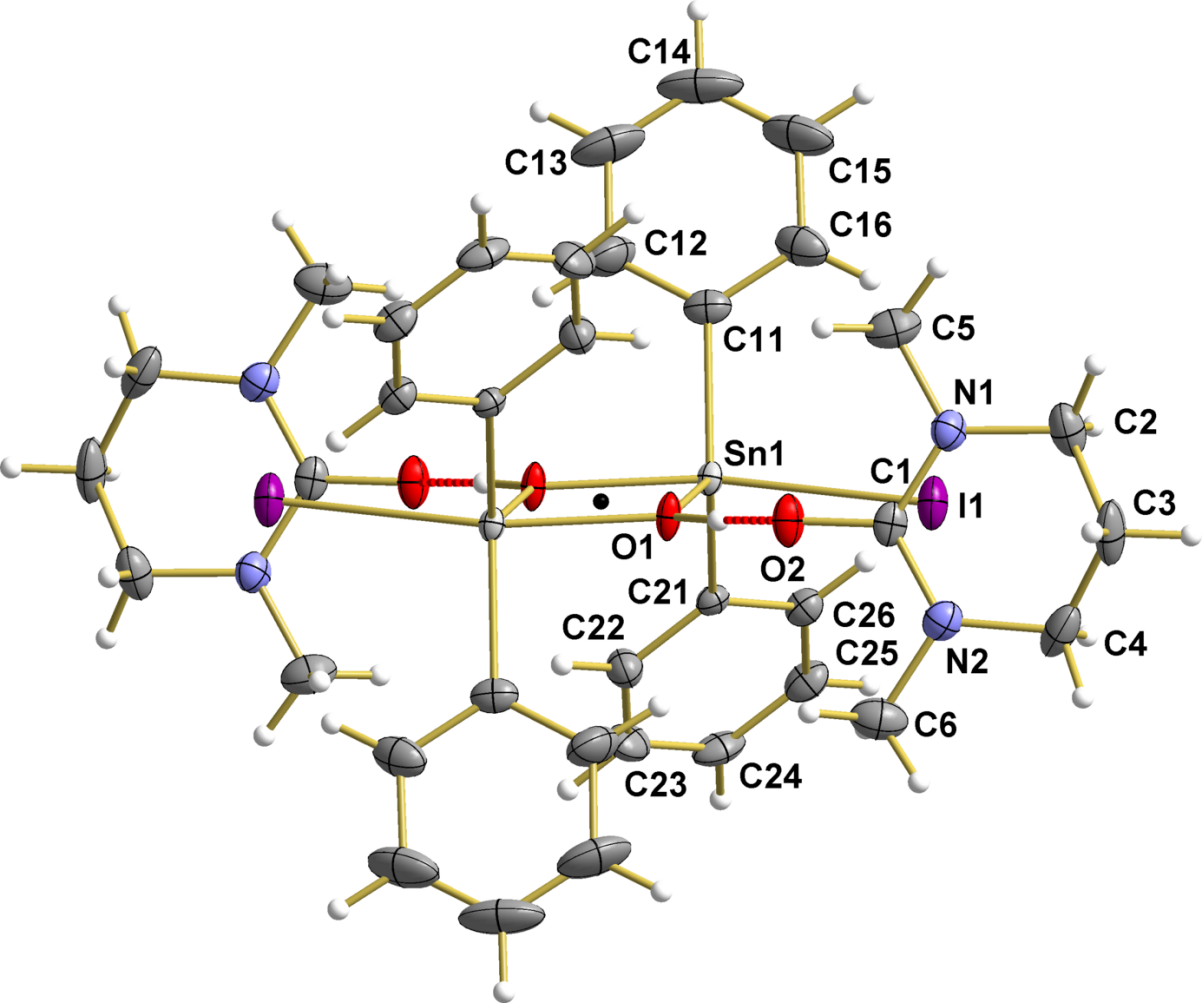
The dimeric, centrosymmetric aggregates found in the crystal of [Ph_2_Sn(OH)I]_2_·2DMPU, showing the atom numbering of the asymmetric unit. With the exception of the hydrogen atoms, which are shown as spheres of arbitrary radius, all other atoms are drawn with displacement ellipsoids at the 40% probability level. Inter­molecular O—H⋯O hydrogen bonds are indicated by dashed sticks in red, black dot = inversion center.

Diorganotin(IV)-hydroxide-halides, *R*_2_Sn(OH)*Hal*, which are the first hydrolysis products of diorganotin(IV) dihalides, *R*_2_Sn*Hal*_2_, are usually difficult to isolate because of further condensation and aggregation reactions resulting in the formation of different kinds of so-called *tetra­organodistannoxanes* like (*R*_2_Sn*Hal*)_2_O, (*R*_2_Sn*Hal*)O(*R*_2_SnOH), and (*R*_2_SnOH)_2_O, all dimeric in the solid state. Structures of pure diorganotin(IV)-hydroxide-halides are only known for *R* = *p*-tolyl and *Hal* = Br (Lo & Ng, 2009 ▸) as well as for *R* = ^*t*^Bu and *Hal* = F, Cl, Br (Puff *et al.*, 1985 ▸), *Hal* = Cl (Di Nicola *et al.*, 2011 ▸), and *Hal* = I (Reuter, 2023 ▸). There are also corresponding compounds in combination with hydrogen bonded Brønstedt bases (*BB*) like *R* = Ph, *Hal* = Cl, *BB* = EtOH (Barba *et al.*, 2007 ▸), *BB* = quinoline (Anacona *et al.*, 2003 ▸) and *R* = ^*t*^Bu, *Hal* = I, *BB* = DMSO (Reuter & Wilberts, 2014 ▸). The title compound, Ph_2_Sn(OH)I]_2_·2DMPU (DMPU is *N*,*N*′-di­methyl­propyl­ene urea,) (Fig. 1 ▸), represents the second example in the class of dimeric diorganotin(IV)-hydroxide-halide solvates [*R*_2_Sn(OH)*Hal*]_2_·2*BB* and *Hal* = I. A few single crystals of this compound, which was probably formed by reaction with atmospheric moisture, were found by chance in a preparation in which the formation of a 1:2 complex between Ph_2_SnI_2_ and DMPU was actually planned.

**Figure 2:**
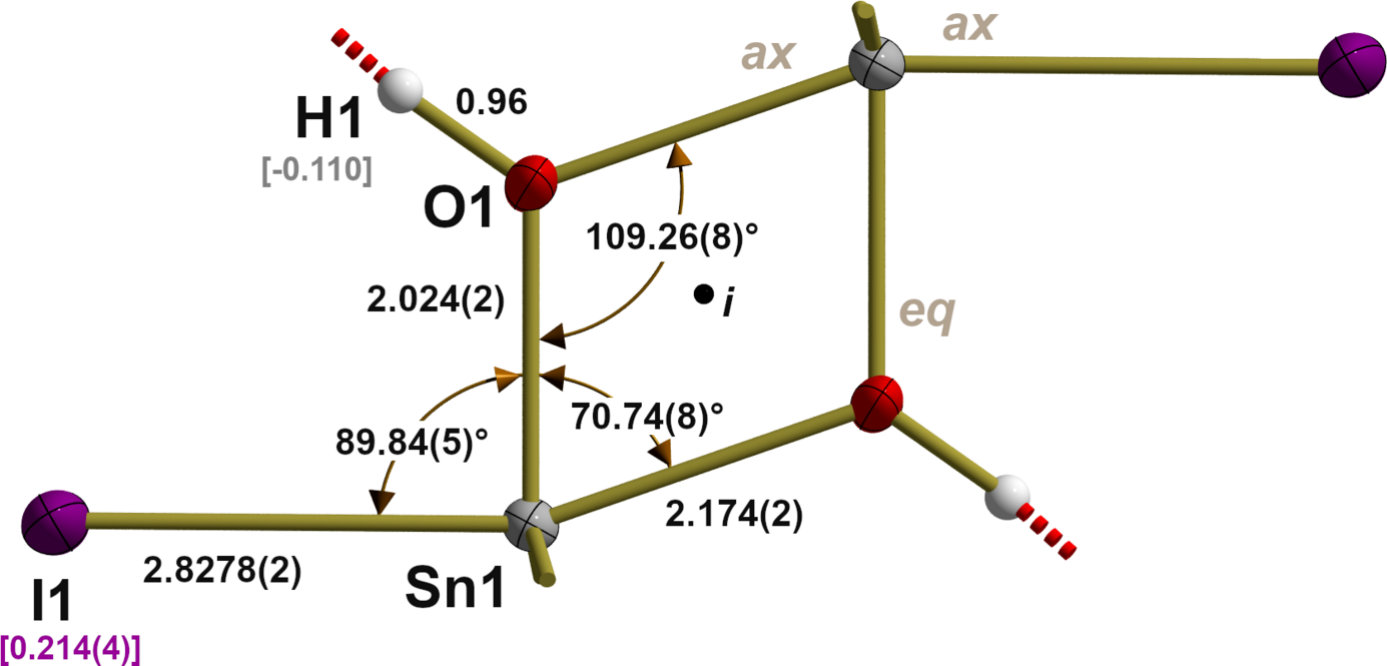
Ball-and-stick model (*i* = inversion center) of the inorganic framework of the [Ph_2_Sn(OH)I]_2_·2DMPU aggregates with selected bond lengths (Å), angles (°) and distances of iodine and hydrogen atoms from the plane of the tin and oxygen atoms in square brackets. Positions of oxygen and iodine atoms within the trigonal–bipyramidal coordination of the tin atoms are labeled by use of the abbreviation *ax* (= axial) and *eq* (= equatorial). For clarity, Ph groups are stripped down to the Sn—C bonds drawn as shortened sticks. Inter­molecular O—H⋯O hydrogen bonds are indicated by dashed sticks in red.

Dimerization takes place according to the same principles as with the previously known compounds *via* OH bridges between two fivefold, trigonal–pyramidal coordinated tin atoms. In the resulting centrosymmetric and therefore planar, four-membered Sn—O ring (Fig. 2 ▸), the angles are obtuse [109.26 (8)°] at the oxygen atoms and acute [70.74 (8)°] at the tin atoms. Tin–oxygen distances differ depending on whether the OH group at the tin atom in question occupies an *equatorial* [2.024 (2) Å] or *axial* position [2.172 (4) Å]. All these values are within the range of the previously determined structures and thus once again confirm the rigidity of this kind of tin–oxygen framework.

The tin–iodine distance [2.8278 (2) Å] is longer than the sum [2.78 Å] of the covalent radii (Cordero *et al.*, 2008 ▸) of tin [1.39 Å] and iodine [1.39 Å] due to the *axial* position of the iodine atom but is shorter than the Sn—I distances in the solvent-free [2.8734 (2) Å] and dmso solvate [2.8852 (2) Å] of the *t*-butyl compound. The position of the iodine atoms is somewhat outside [±0.278 Å] the plane of the four-membered Sn—O ring. In the case of the two phenyl groups which are in *equatorial* positions the tin–carbon distances of 2.139 (3) and 2.141 (2) Å are somewhat larger than the tin-carbon distances in the previously mentioned *hydroxide-halides* with *R* = Ph [2.119 (3)–2.134 (3) Å, mean value = 2.120 (8) Å].

The phenyl groups do not exhibit any major structural peculiarities, even if they show strong thermal movement, especially in the case of the first one (C11–C16). They are almost planar with greater [Δ = ±0.010 (3) Å] deviations Δ in terms of the distance of the C atoms from the least-squares plane in the first than in the second one [Δ = ±0.004 (2) Å]. The carbon–carbon distances vary from 1.347 (7)–1.402 (5) Å in the first and 1.375 (5)–1.396 (4) Å in the second phenyl group but their mean value of 1.384 (16) Å is in good agreement with the value of 1.380 (13) Å given by Allen *et al.* (1987 ▸) for this kind of aromatic C—C bonds in phenyl groups. Among the bond angles [118.9 (3)–121.3 (4)°] those at the *ipso*-carbon atoms are the smallest one [mean value: 119.1 (2)°] in accordance with the so-called *ipso*-effect (Jones, 1988 ▸).

**Figure 3:**
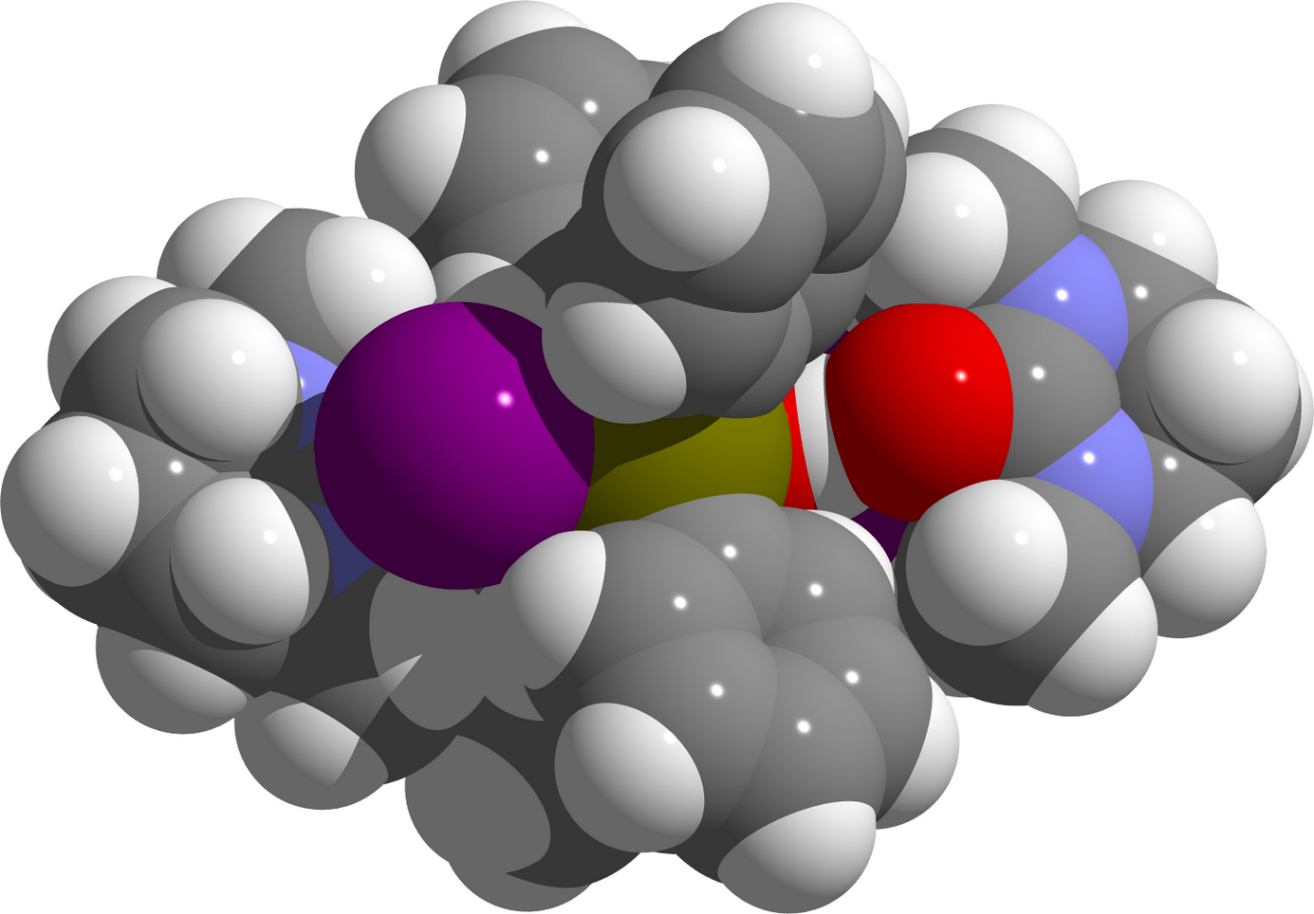
Space-filling model of the [Ph_2_Sn(OH)I]_2_·2DMPU aggregates viewed edge-on to the four-membered Sn—O ring and the inter­molecular O—H⋯O hydrogen bonds. Color code of the atoms: I = violet, H = white, C = gray, O = red, Sn = brass.

*N*,*N*′-Di­methyl­propyl­ene urea, DMPU, is a polar aprotic solvent that is often used in organic synthesis as a substitute for the carcinogenic hexa­methyl­phospho­ric acid tri­amide, HMPTA (Mukhopadhyay & Seebach, 1982 ▸). In crystal structures, it is often found as a complex ligand, co-crystallizate or hydrogen-bonded Lewis base. Typical examples are [Nd(dmpu)_6_]I_3_·3DMPU (Lundberg *et al.*, 2010 ▸) and [(^*i*^PrSn)_12_O_14_(OH)_6_]Cl_2_·4DMPU·4H_2_O (Puff & Reuter, 1989 ▸). In the present structure, the DMPU mol­ecule is connected as hydrogen acceptor with the hydroxyl group of the *hydroxide-halide* as hydrogen donor. The structural parameters of this cyclic urea derivative are strongly influenced by the urea building unit with almost trigonal–planar-coordinated carbon and nitro­gen atoms. Thus, the carbon–nitro­gen distances are 1.351 (4)/1.355 (3) Å in the case of the carbon atom of the carbonyl group and 1.452 (4), 1.456 (4) Å in the case of the tetra­hedrally coordinated carbon atoms while the endocyclic bond angles at the nitro­gen atoms reach 122.2 (2)/122.9 (2)°. The endocyclic carbon–carbon bond lengths are somewhat shorter [1.501 (5)/1.515 (5) Å] than a typical C—C single bond [1.524 (14) Å; Allen *et al.*, 1987 ▸] between *sp*^3^-hybridized carbon atoms, but the bond angles [109.4 (3)-109.8 (3)°] correspond very well to this kind of hybridization. The exocyclic C—O bond length of 1.257 (3) Å is slightly elongated in comparison with a C—O double bond between a *sp*^2^-hybridized carbon atom and an oxygen atom. Elongation is probably due to the formation of the hydrogen bond with the OH group of the *hydroxide-halide*. This hydrogen bond is quite strong as the donor–acceptor distances and the nearly linear alignment indicate (Table 1 ▸). As a result of these hydrogen bonds, each strongly polar bond of one mol­ecule is shielded by the apolar parts of the other mol­ecule (Fig. 3 ▸), and the inter­actions between the adducts are limited to van der Waals bonds.

## Synthesis and crystallization

Slightly yellowish, block-shaped single crystals of the title compound were found in a micro-scale experiment (Schröder *et al.*, 2024 ▸) in which the 1:2 complex of di­phenyl­tin(IV) iodide, Ph_2_SnI_2_, with DMPU should be formed in 96% ethanol as solvent. After prolonged exposure to air, the alcohol had completely evaporated. In the remaining sticky residue, instead of the desired complex, only numerous crystals of the title compound were found, which had probably formed through contact with moist air.

## Refinement

Crystal data, data collection and structure refinement details are summarized in Table 2 ▸. The positions of all H atoms were clearly identified in difference-Fourier syntheses. Hydrogen atoms attached to carbon atoms were refined with calculated positions (–CH_3_ = 0.98 Å, –CH_2_– = 0.99 Å, –CH_arom_– = 0.95 Å) and common *U*_iso_(H) parameters for all hydrogen atoms of the DMPU mol­ecule and one for each of the hydrogen atoms of the two phenyl groups. In order to obtain a realistic description of the hydrogen bond, the maximum electron density resulting from the X-ray data for the hydrogen atom was used to determine the direction of the O—H bond, while the position of the nucleus of the hydrogen atom was calculated using an O—H distance in better accordance with gas phase and neutron diffraction data. For this purpose, the position of the H atom of the hydroxyl group was refined with a fixed O—H distance of 0.96 Å before it was fixed and allowed to ride on the parent oxygen atom with a *U*_iso_(H) parameter.

## Supplementary Material

Crystal structure: contains datablock(s) I. DOI: 10.1107/S2414314625007114/zl4086sup1.cif

Structure factors: contains datablock(s) I. DOI: 10.1107/S2414314625007114/zl4086Isup2.hkl

CCDC reference: 2478813

Additional supporting information:  crystallographic information; 3D view; checkCIF report

## Figures and Tables

**Table 1:** Hydrogen-bond geometry (Å, °)

*D*—H⋯*A*	*D*—H	H⋯*A*	*D*⋯*A*	*D*—H⋯*A*
O1—H1⋯O2	0.96	1.63	2.584 (3)	172

**Table 2:** Experimental details

Crystal data	
Chemical formula	[Sn(C_6_H_5_)_2_I(OH)]·2C_6_H_12_N_2_O
*M*_r_	1089.95
Crystal system, space group	Monoclinic, *P*2_1_/*n*
Temperature (K)	100
*a*, *b*, *c* (Å)	10.1014 (4), 11.3064 (4), 17.1982 (7)
β (°)	93.027 (2)
*V* (Å^3^)	1961.47 (13)
*Z*	2
Radiation type	Mo *K*α
μ (mm^−1^)	2.89
Crystal size (mm)	0.30 × 0.18 × 0.09

Data collection	
Diffractometer	Bruker APEXII CCD
Absorption correction	Multi-scan (*SADABS*; Krause *et al.*, 2015 ▸)
*T*_min_, *T*_max_	0.482, 0.671
No. of measured, independent and observed [*I* > 2σ(*I*)] reflections	100676, 4729, 4323
*R*_int_	0.079
(sin θ/λ)_max_ (Å^−1^)	0.661

Refinement	
*R*[*F*^2^ > 2σ(*F*^2^)], *wR*(*F*^2^), *S*	0.024, 0.061, 1.05
No. of reflections	4729
No. of parameters	225
H-atom treatment	H-atom parameters constrained
Δρ_max_, Δρ_min_ (e Å^−3^)	1.25, −1.47

Computer programs: *APEX2* and *SAINT* (Bruker, 2009 ▸), *SHELXS97* (Sheldrick 2008 ▸), *SHELXL2014/7* (Sheldrick, 2015 ▸), *DIAMOND* (Brandenburg, 2006 ▸), *Mercury* (Macrae *et al.* (2020 ▸) and *publCIF* (Westrip, 2010 ▸).

## full crystallographic data

### Di-µ-hydroxido-bis[iodidodiphenyltin(IV)]–1,3-dimethylimidazolidin-2-one (1/2) Crystal data

**Table:**

[Sn(C_6_H_5_)_2_I(OH)]·2C_6_H_12_N_2_O	*F*(000) = 1056
*M**_r_* = 1089.95	*D*_x_ = 1.845 Mg m^−^^3^
Monoclinic, *P*2_1_/*n*	Mo *K*α radiation, λ = 0.71073 Å
*a* = 10.1014 (4) Å	Cell parameters from 9764 reflections
*b* = 11.3064 (4) Å	θ = 2.7–29.2°
*c* = 17.1982 (7) Å	µ = 2.89 mm^−^^1^
β = 93.027 (2)°	*T* = 100 K
*V* = 1961.47 (13) Å^3^	Spat, colourless
*Z* = 2	0.30 × 0.18 × 0.09 mm

### Di-µ-hydroxido-bis[iodidodiphenyltin(IV)]–1,3-dimethylimidazolidin-2-one (1/2) Data collection

**Table:**

Bruker APEXII CCD diffractometer	4323 reflections with *I* > 2σ(*I*)
φ and ω scans	*R*_int_ = 0.079
Absorption correction: multi-scan (SADABS; Krause *et al.*, 2015)	θ_max_ = 28.0°, θ_min_ = 2.7°
*T*_min_ = 0.482, *T*_max_ = 0.671	*h* = −13→13
100676 measured reflections	*k* = −14→14
4729 independent reflections	*l* = −22→22

### Di-µ-hydroxido-bis[iodidodiphenyltin(IV)]–1,3-dimethylimidazolidin-2-one (1/2) Refinement

**Table:**

Refinement on *F*^2^	Hydrogen site location: mixed
Least-squares matrix: full	H-atom parameters constrained
*R*[*F*^2^ > 2σ(*F*^2^)] = 0.024	*w* = 1/[σ^2^(*F*_o_^2^) + (0.0239*P*)^2^ + 3.8824*P*] where *P* = (*F*_o_^2^ + 2*F*_c_^2^)/3
*wR*(*F*^2^) = 0.061	(Δ/σ)_max_ = 0.001
*S* = 1.05	Δρ_max_ = 1.25 e Å^−^^3^
4729 reflections	Δρ_min_ = −1.47 e Å^−^^3^
225 parameters	Extinction correction: *SHELXL2014/7* (Sheldrick, 2015), Fc^*^=kFc[1+0.001xFc^2^λ^3^/sin(2θ)]^-1/4^
0 restraints	Extinction coefficient: 0.00232 (17)
Primary atom site location: structure-invariant direct methods	

### Di-µ-hydroxido-bis[iodidodiphenyltin(IV)]–1,3-dimethylimidazolidin-2-one (1/2) Special details

**Table:**

Geometry. All esds (except the esd in the dihedral angle between two l.s. planes) are estimated using the full covariance matrix. The cell esds are taken into account individually in the estimation of esds in distances, angles and torsion angles; correlations between esds in cell parameters are only used when they are defined by crystal symmetry. An approximate (isotropic) treatment of cell esds is used for estimating esds involving l.s. planes.

### Di-µ-hydroxido-bis[iodidodiphenyltin(IV)]–1,3-dimethylimidazolidin-2-one (1/2) Fractional atomic coordinates and isotropic or equivalent isotropic displacement parameters (Å^2^)

**Table:**

	*x*	*y*	*z*	*U*_iso_*/*U*_eq_	
Sn1	0.04841 (2)	0.35489 (2)	0.49934 (2)	0.01348 (6)	
C11	0.1816 (3)	0.3343 (2)	0.59931 (16)	0.0228 (6)	
C12	0.1508 (4)	0.3855 (4)	0.66929 (19)	0.0417 (8)	
H12	0.0736	0.4329	0.6721	0.063 (6)*	
C13	0.2341 (5)	0.3668 (4)	0.7360 (2)	0.0582 (12)	
H13	0.2112	0.4003	0.7841	0.063 (6)*	
C14	0.3459 (5)	0.3024 (4)	0.7332 (3)	0.0596 (13)	
H14	0.4016	0.2911	0.7788	0.063 (6)*	
C15	0.3787 (4)	0.2530 (3)	0.6634 (3)	0.0536 (12)	
H15	0.4580	0.2082	0.6609	0.063 (6)*	
C16	0.2964 (3)	0.2684 (3)	0.5965 (2)	0.0351 (7)	
H16	0.3193	0.2335	0.5488	0.063 (6)*	
C21	−0.1222 (2)	0.2451 (2)	0.47739 (14)	0.0156 (5)	
C22	−0.2502 (3)	0.2881 (3)	0.48144 (17)	0.0253 (6)	
H22	−0.2645	0.3695	0.4917	0.031 (4)*	
C23	−0.3580 (3)	0.2115 (3)	0.47049 (19)	0.0325 (7)	
H23	−0.4456	0.2413	0.4730	0.031 (4)*	
C24	−0.3383 (3)	0.0930 (3)	0.45601 (16)	0.0290 (6)	
H24	−0.4121	0.0414	0.4489	0.031 (4)*	
C25	−0.2116 (3)	0.0499 (2)	0.45184 (17)	0.0260 (6)	
H25	−0.1980	−0.0318	0.4419	0.031 (4)*	
C26	−0.1032 (3)	0.1254 (2)	0.46211 (16)	0.0206 (5)	
H26	−0.0159	0.0952	0.4587	0.031 (4)*	
I1	0.19964 (2)	0.24368 (2)	0.38647 (2)	0.02578 (7)	
O1	0.06719 (18)	0.51134 (15)	0.44382 (11)	0.0193 (4)	
H1	0.1273	0.5266	0.4036	0.048 (11)*	
O2	0.2304 (2)	0.57144 (17)	0.33999 (12)	0.0253 (4)	
C1	0.2989 (3)	0.5083 (2)	0.29735 (15)	0.0187 (5)	
N1	0.4246 (2)	0.4779 (2)	0.32074 (14)	0.0227 (5)	
C2	0.5013 (3)	0.3930 (3)	0.27814 (19)	0.0321 (7)	
H2A	0.4822	0.3118	0.2960	0.049 (5)*	
H2B	0.5972	0.4084	0.2882	0.049 (5)*	
C3	0.4654 (4)	0.4039 (3)	0.19181 (19)	0.0406 (8)	
H3A	0.4939	0.4820	0.1727	0.049 (5)*	
H3B	0.5116	0.3419	0.1630	0.049 (5)*	
C4	0.3182 (4)	0.3907 (3)	0.17794 (18)	0.0366 (8)	
H4A	0.2932	0.4055	0.1224	0.049 (5)*	
H4B	0.2915	0.3089	0.1906	0.049 (5)*	
N2	0.2502 (2)	0.4741 (2)	0.22624 (13)	0.0240 (5)	
C5	0.4672 (3)	0.5010 (3)	0.40127 (19)	0.0352 (7)	
H5A	0.4406	0.5811	0.4156	0.047 (4)*	
H5B	0.5639	0.4939	0.4075	0.047 (4)*	
H5C	0.4260	0.4434	0.4351	0.047 (4)*	
C6	0.1125 (3)	0.5007 (3)	0.2053 (2)	0.0370 (7)	
H6A	0.0554	0.4447	0.2313	0.047 (4)*	
H6B	0.0968	0.4938	0.1488	0.047 (4)*	
H6C	0.0923	0.5814	0.2217	0.047 (4)*	

### Di-µ-hydroxido-bis[iodidodiphenyltin(IV)]–1,3-dimethylimidazolidin-2-one (1/2) Atomic displacement parameters (Å^2^)

**Table:**

	*U*^11^	*U*^22^	*U*^33^	*U*^12^	*U*^13^	*U*^23^
Sn1	0.01511 (9)	0.01051 (9)	0.01513 (10)	−0.00128 (6)	0.00380 (6)	−0.00111 (6)
C11	0.0244 (14)	0.0204 (12)	0.0231 (14)	−0.0079 (10)	−0.0037 (11)	0.0057 (10)
C12	0.0402 (19)	0.062 (2)	0.0223 (16)	−0.0025 (17)	−0.0032 (14)	−0.0060 (15)
C13	0.069 (3)	0.080 (3)	0.0241 (18)	−0.018 (2)	−0.0147 (18)	0.0021 (18)
C14	0.066 (3)	0.057 (3)	0.051 (3)	−0.022 (2)	−0.037 (2)	0.026 (2)
C15	0.051 (2)	0.037 (2)	0.069 (3)	0.0016 (16)	−0.031 (2)	0.0154 (18)
C16	0.0325 (17)	0.0232 (14)	0.048 (2)	−0.0008 (12)	−0.0114 (15)	0.0045 (13)
C21	0.0155 (11)	0.0177 (12)	0.0136 (11)	−0.0031 (9)	0.0008 (9)	−0.0007 (9)
C22	0.0220 (13)	0.0242 (14)	0.0299 (15)	0.0010 (11)	0.0015 (11)	−0.0035 (11)
C23	0.0158 (13)	0.0473 (19)	0.0341 (17)	−0.0017 (13)	0.0003 (12)	−0.0062 (14)
C24	0.0265 (14)	0.0402 (17)	0.0200 (14)	−0.0177 (13)	−0.0009 (11)	−0.0012 (12)
C25	0.0334 (15)	0.0193 (13)	0.0253 (14)	−0.0091 (11)	0.0010 (11)	−0.0014 (11)
C26	0.0240 (13)	0.0170 (12)	0.0209 (13)	−0.0036 (10)	0.0014 (10)	0.0006 (10)
I1	0.02964 (11)	0.01696 (10)	0.03231 (12)	−0.00113 (7)	0.01641 (8)	−0.00387 (7)
O1	0.0276 (10)	0.0116 (8)	0.0200 (9)	0.0011 (7)	0.0144 (7)	0.0004 (7)
O2	0.0298 (10)	0.0187 (9)	0.0290 (10)	−0.0001 (8)	0.0167 (8)	0.0010 (8)
C1	0.0237 (13)	0.0137 (11)	0.0197 (13)	−0.0041 (10)	0.0103 (10)	0.0027 (9)
N1	0.0224 (11)	0.0246 (11)	0.0217 (11)	−0.0011 (9)	0.0053 (9)	−0.0003 (9)
C2	0.0331 (16)	0.0292 (15)	0.0353 (17)	0.0098 (13)	0.0138 (13)	0.0053 (13)
C3	0.058 (2)	0.0357 (18)	0.0309 (17)	0.0173 (16)	0.0256 (16)	0.0019 (14)
C4	0.062 (2)	0.0260 (15)	0.0218 (15)	0.0044 (15)	0.0054 (14)	−0.0058 (12)
N2	0.0285 (12)	0.0230 (11)	0.0207 (11)	−0.0008 (9)	0.0029 (9)	0.0021 (9)
C5	0.0366 (17)	0.0389 (18)	0.0294 (16)	−0.0106 (14)	−0.0051 (13)	0.0016 (13)
C6	0.0303 (16)	0.0420 (18)	0.0381 (18)	−0.0072 (14)	−0.0043 (13)	0.0089 (14)

### Di-µ-hydroxido-bis[iodidodiphenyltin(IV)]–1,3-dimethylimidazolidin-2-one (1/2) Geometric parameters (Å, º)

**Table:**

Sn1—O1	2.024 (2)	C25—H25	0.9500
Sn1—C11	2.139 (3)	C26—H26	0.9500
Sn1—C21	2.141 (2)	O1—Sn1^i^	2.174 (2)
Sn1—O1^i^	2.174 (2)	O1—H1	0.9599
Sn1—I1	2.8278 (2)	O2—C1	1.257 (3)
C11—C16	1.382 (4)	C1—N2	1.351 (4)
C11—C12	1.385 (5)	C1—N1	1.355 (3)
C12—C13	1.402 (5)	N1—C5	1.452 (4)
C12—H12	0.9500	N1—C2	1.456 (4)
C13—C14	1.347 (7)	C2—C3	1.515 (5)
C13—H13	0.9500	C2—H2A	0.9900
C14—C15	1.380 (7)	C2—H2B	0.9900
C14—H14	0.9500	C3—C4	1.501 (5)
C15—C16	1.395 (5)	C3—H3A	0.9900
C15—H15	0.9500	C3—H3B	0.9900
C16—H16	0.9500	C4—N2	1.454 (4)
C21—C22	1.387 (4)	C4—H4A	0.9900
C21—C26	1.393 (3)	C4—H4B	0.9900
C22—C23	1.396 (4)	N2—C6	1.450 (4)
C22—H22	0.9500	C5—H5A	0.9800
C23—C24	1.378 (5)	C5—H5B	0.9800
C23—H23	0.9500	C5—H5C	0.9800
C24—C25	1.375 (4)	C6—H6A	0.9800
C24—H24	0.9500	C6—H6B	0.9800
C25—C26	1.393 (4)	C6—H6C	0.9800
			
O1—Sn1—C11	113.80 (9)	C25—C26—H26	119.9
O1—Sn1—C21	121.22 (9)	C21—C26—H26	119.9
C11—Sn1—C21	123.01 (10)	Sn1—O1—Sn1^i^	109.26 (8)
O1—Sn1—O1^i^	70.74 (8)	Sn1—O1—H1	125.2
C11—Sn1—O1^i^	92.52 (10)	Sn1^i^—O1—H1	125.0
C21—Sn1—O1^i^	92.09 (8)	O2—C1—N2	120.4 (3)
O1—Sn1—I1	89.84 (5)	O2—C1—N1	120.4 (3)
C11—Sn1—I1	99.43 (8)	N2—C1—N1	119.1 (2)
C21—Sn1—I1	94.58 (7)	C1—N1—C5	117.6 (2)
O1^i^—Sn1—I1	160.12 (5)	C1—N1—C2	122.2 (2)
C16—C11—C12	118.9 (3)	C5—N1—C2	117.3 (3)
C16—C11—Sn1	121.6 (2)	N1—C2—C3	109.5 (2)
C12—C11—Sn1	119.5 (2)	N1—C2—H2A	109.8
C11—C12—C13	119.7 (4)	C3—C2—H2A	109.8
C11—C12—H12	120.2	N1—C2—H2B	109.8
C13—C12—H12	120.2	C3—C2—H2B	109.8
C14—C13—C12	121.3 (4)	H2A—C2—H2B	108.2
C14—C13—H13	119.3	C4—C3—C2	109.4 (3)
C12—C13—H13	119.3	C4—C3—H3A	109.8
C13—C14—C15	119.4 (3)	C2—C3—H3A	109.8
C13—C14—H14	120.3	C4—C3—H3B	109.8
C15—C14—H14	120.3	C2—C3—H3B	109.8
C14—C15—C16	120.4 (4)	H3A—C3—H3B	108.2
C14—C15—H15	119.8	N2—C4—C3	109.8 (3)
C16—C15—H15	119.8	N2—C4—H4A	109.7
C11—C16—C15	120.2 (4)	C3—C4—H4A	109.7
C11—C16—H16	119.9	N2—C4—H4B	109.7
C15—C16—H16	119.9	C3—C4—H4B	109.7
C22—C21—C26	119.2 (2)	H4A—C4—H4B	108.2
C22—C21—Sn1	122.11 (19)	C1—N2—C6	117.7 (3)
C26—C21—Sn1	118.62 (19)	C1—N2—C4	122.9 (2)
C21—C22—C23	119.9 (3)	C6—N2—C4	118.0 (3)
C21—C22—H22	120.1	N1—C5—H5A	109.5
C23—C22—H22	120.1	N1—C5—H5B	109.5
C24—C23—C22	120.5 (3)	H5A—C5—H5B	109.5
C24—C23—H23	119.7	N1—C5—H5C	109.5
C22—C23—H23	119.7	H5A—C5—H5C	109.5
C25—C24—C23	119.8 (3)	H5B—C5—H5C	109.5
C25—C24—H24	120.1	N2—C6—H6A	109.5
C23—C24—H24	120.1	N2—C6—H6B	109.5
C24—C25—C26	120.2 (3)	H6A—C6—H6B	109.5
C24—C25—H25	119.9	N2—C6—H6C	109.5
C26—C25—H25	119.9	H6A—C6—H6C	109.5
C25—C26—C21	120.3 (3)	H6B—C6—H6C	109.5
			
C16—C11—C12—C13	−1.8 (5)	Sn1—C21—C26—C25	176.4 (2)
Sn1—C11—C12—C13	176.5 (3)	O2—C1—N1—C5	11.5 (4)
C11—C12—C13—C14	1.7 (6)	N2—C1—N1—C5	−171.3 (2)
C12—C13—C14—C15	−0.5 (7)	O2—C1—N1—C2	172.0 (2)
C13—C14—C15—C16	−0.7 (6)	N2—C1—N1—C2	−10.8 (4)
C12—C11—C16—C15	0.6 (5)	C1—N1—C2—C3	34.0 (4)
Sn1—C11—C16—C15	−177.6 (3)	C5—N1—C2—C3	−165.5 (3)
C14—C15—C16—C11	0.6 (6)	N1—C2—C3—C4	−54.7 (4)
C26—C21—C22—C23	0.2 (4)	C2—C3—C4—N2	53.8 (3)
Sn1—C21—C22—C23	−176.8 (2)	O2—C1—N2—C6	−7.2 (4)
C21—C22—C23—C24	0.3 (5)	N1—C1—N2—C6	175.6 (2)
C22—C23—C24—C25	−0.4 (5)	O2—C1—N2—C4	−173.0 (3)
C23—C24—C25—C26	−0.1 (4)	N1—C1—N2—C4	9.8 (4)
C24—C25—C26—C21	0.6 (4)	C3—C4—N2—C1	−32.4 (4)
C22—C21—C26—C25	−0.6 (4)	C3—C4—N2—C6	161.8 (3)

Symmetry code: (i) −*x*, −*y*+1, −*z*+1.

### Di-µ-hydroxido-bis[iodidodiphenyltin(IV)]–1,3-dimethylimidazolidin-2-one (1/2) Hydrogen-bond geometry (Å, º)

**Table:**

*D*—H···*A*	*D*—H	H···*A*	*D*···*A*	*D*—H···*A*
O1—H1···O2	0.96	1.63	2.584 (3)	172
